# An empirical assay for assessing genomic sensitivity and for improving cancer diagnostics

**DOI:** 10.1186/1755-8166-7-S1-I7

**Published:** 2014-01-21

**Authors:** Diana Anderson

**Affiliations:** 1 University of Bradford, Richmond Road, Bradford BD7 1DP, UK

## 

Detection tests have been developed for many cancers, but there is no single test to identify cancer in general. We have developed such an assay. In this modified patented Comet assay, we investigated lymphocytes of 208 individuals: 20 melanoma, 34 colon cancer, 4 lung cancer patients, 18 suspect melanoma, 28 polyposis, 10 COPD patients and 94 healthy volunteers. The natural logarithm of the Olive tail moment was plotted for exposure to UVA through different agar depths for each of the above groups and analyzed using a repeated measures regression model. Response patterns for cancer patients formed a plateau after treating with UVA where intensity varied with different agar depths. In comparison, response patterns for healthy individuals returned towards control values and for pre/suspected cancers, were intermediate with less of a plateau. All cancers tested exhibited comparable responses. Analyses of Receiver Operating Characteristic curves, of mean log Olive tail moments, for all cancers plus pre/suspected-cancer versus controls gave a value for the area under the curve of 0.87; for cancer versus pre/suspected-cancer plus controls the value was 0.89; and for cancer alone versus controls alone (excluding pre/suspected-cancer), the value was 0.93. By varying the threshold for test positivity, its sensitivity or specificity can approach 100% whilst maintaining acceptable complementary measures. Evidence presented indicates that this modified assay shows promise as both a stand-alone test and as a possible adjunct to other investigative procedures, as part of detection programs for a range of cancers.

